# Support Vector Machine Classification of Obsessive-Compulsive Disorder Based on Whole-Brain Volumetry and Diffusion Tensor Imaging

**DOI:** 10.3389/fpsyt.2018.00524

**Published:** 2018-10-23

**Authors:** Cong Zhou, Yuqi Cheng, Liangliang Ping, Jian Xu, Zonglin Shen, Linling Jiang, Li Shi, Shuran Yang, Yi Lu, Xiufeng Xu

**Affiliations:** ^1^Department of Psychiatry, First Affiliated Hospital of Kunming Medical University, Kunming, China; ^2^Postgraduate College, Kunming Medical University, Kunming, China; ^3^Department of Internal Medicine, First Affiliated Hospital of Kunming Medical University, Kunming, China; ^4^Department of Medical Imaging, First Affiliated Hospital of Kunming Medical University, Kunming, China

**Keywords:** obsessive-compulsive disorder, support vector machine, structural magnetic resonance imaging, brain volumetry, diffusion tensor imaging

## Abstract

Magnetic resonance imaging (MRI) methods have been used to detect cerebral anatomical distinction between obsessive-compulsive disorder (OCD) patients and healthy controls (HC). Machine learning approach allows for the possibility of discriminating patients on the individual level. However, few studies have used this automatic technique based on multiple modalities to identify potential biomarkers of OCD. High-resolution structural MRI and diffusion tensor imaging (DTI) data were acquired from 48 OCD patients and 45 well-matched HC. Gray matter volume (GMV), white matter volume (WMV), fractional anisotropy (FA), and mean diffusivity (MD) were extracted as four features were examined using support vector machine (SVM). Ten brain regions of each feature contributed most to the classification were also estimated. Using different algorithms, the classifier achieved accuracies of 72.08, 61.29, 80.65, and 77.42% for GMV, WMV, FA, and MD, respectively. The most discriminative gray matter regions that contributed to the classification were mainly distributed in the orbitofronto-striatal “affective” circuit, the dorsolateral, prefronto-striatal “executive” circuit and the cerebellum. For WMV feature and the two feature sets of DTI, the shared regions contributed the most to the discrimination mainly included the uncinate fasciculus, the cingulum in the hippocampus, corticospinal tract, as well as cerebellar peduncle. Based on whole-brain volumetry and DTI images, SVM algorithm revealed high accuracies for distinguishing OCD patients from healthy subjects at the individual level. Computer-aided method is capable of providing accurate diagnostic information and might provide a new perspective for clinical diagnosis of OCD.

## Introduction

The core characteristics of obsessive-compulsive disorder (OCD) are intrusive recurrent thoughts and/or repetitive behaviors. Abnormalities of gray matter (GM) and white matter (WM) microstructures are considered to be related with its neural pathogenesis. Nowadays magnetic resonance imaging (MRI) approaches provide a perspective to investigate the neuropathological changes of OCD and allow researchers to identify better biological markers of this disease. Voxel-based morphometry (VBM) analysis allows the investigation of gray matter volume (GMV) and white matter volume (WMV) in the whole brain (1). Diffusion tensor imaging (DTI), on the other hand, is available to measure the *in vivo* water molecule diffusion within the WM fibers, which renders more exquisite details on microstructure changes in WM (2). Fractional anisotropy (FA) and mean diffusivity (MD) are the two most widely used diffusion indices to investigate the pathology of OCD (3, 4).

To date numerous neuroimaging studies have used the between—group comparison—types of analyses to investigate subtle differences between OCD patients and healthy controls (HC). Reported volumetric abnormalities lied in multiple neural structures. Volume reduction in the medial orbitofrontal, anterior cingulate and temporolimbic cortices, and tissue expansion in the striatum and thalamus was among the most widely accepted pathological model of OCD which assumes brain abnormalities in the “affective circuit” (1, 5, 6). Additionally, volume changes of the cortical source of the dorsolateral (DL) prefronto-striatal “executive” circuit (dorsomedial, DL, ventrolateral and frontopolar prefrontal cortices), and of reciprocally connected regions (temporo-parieto-occipital associative areas) are consistently described in OCD patients (6). Reported WM integrality abnormalities also involved extensive brain areas, such as the corpus callosum (CC), cingulum bundles, corticospinal tract, superior longitudinal fasciculus (SLF), uncinate fasciculus (UNC), and cerebellum (1, 4). However, the results obtained by various studies were substantially heterogeneous. Neuropsychiatric disorders are usually characterized by indistinct structure abnormalities rather than a significant abnormal region (7). The group-level analysis usually requires large samples and needs to be corrected by strict multiple comparisons, and the alterations are often too small to detect and lack a reliable differentiation between patients and control subjects (8). Furthermore, group-based methods are neither helpful to diagnose patients at the individual level nor to infer specific clinical outcomes for an individual patient (9).

Advances in neuroimaging have resulted in the use of automated techniques for multivariate pattern analysis. The MRI-related machine learning technique offers a systematic approach in developing sophisticated, automatic, and objective classification frameworks for analyzing high-dimensional data and provides promise for improving the sensitivity and/or specificity of detection and diagnosis of disease (10–12). The analysis based on multivariate pattern is more sensitive to identify subtle differences in the brain structure than group-level statistics. Machine learning-based pattern recognition techniques hold the potential to detect biomarkers on the basis of neuroimaging data and make it possible to combine complementary information from different sources efficiently (13). The techniques also enable people to predict the future course of the disease and the response to treatment at the individual patient level (14). Thus, it holds high clinical values. The support vector machine (SVM) pattern recognition has been demonstrated to be useful for MRI classification. The SVM algorithm establishes model by discriminating the different categories (such as patients and controls) and further applying new data to test its generalizability (15, 16). So far, SVM classifications have been successfully applied to a range of MRI modalities aiming to automate the diagnosis of neuropsychiatric disorders in numerous studies (17–29), and the high accuracies were extremely impressive.

To our best knowledge, few studies have used the SVM approach based on MRI data to perform diagnostic or predictive investigations in OCD patients. A recent review of OCD multivariate pattern analysis based on neuroimaging data found that it is able to classify OCD diagnosis with accuracies ranging from 66 up to 100% (30). Among these studies, Li et al. (16) found FA value showed promise for discriminating OCD from healthy controls (HC) using SVM. The study provided the first evidence of a quite high identification rate presented as a sensitivity of 86%, a specificity of 82% and a significant accuracy of 84%. Moreover, they found the white matter regions which contributed the most to such discrimination mainly included bilateral prefrontal and temporal regions, inferior fronto-occipital fasciculus, superior fronto-parietal fasciculus, splenium of corpus callosum, and left middle cingulum bundle. Hu et al. (31) used structural neuroanatomy of GM and WM volume and reported these structural images could accurately discriminate between patients with OCD and HC. They found the classification accuracies for SVM using GM and WM anatomy were all above 75%, and three main distributed networks including the fronto-striatal circuit, the temporo-parieto-occipital junction and the cerebellum provided high discriminative power. Several other OCD studies conducted different feature selection algorithms and revealed a more comprehensive characterization of the disorder, thus yielding a superior identification of OCD patients based on their brain anatomy (32, 33). Besides, in a psychiatric symptoms predicting study, Hoexter et al. (34) employed support vector regression (SVR) in 37 treatment-naïve adult patients to evaluate whether gray matter volumes of the cortical–subcortical loops contain informative value to predict OCD symptom severity. They found the left medial orbitofrontal cortex and the left putamen may identify neurobiological markers to predict OCD symptom severity based on individual structural MRI datasets.

Multimodal neuroimaging features may reflect different aspects of brain tissue and may be a supplement to comprehend the pathological mechanism of OCD (28, 35). Our previous work has found that OCD patients exhibited symptom-related reduced right posterior cingulate cortex cortical thickness, and disrupted WM integrity in the genu and body of corpus callosum (36). Biomarkers from multiple modalities may provide complementary information for the diagnosis of OCD. However, existing MRI-based SVM analysis studies focused on only a single modality of MRI. To further comprehensively and systematically evaluate the SVM approach applied in distinguishing patients with OCD from HC, and also to provide classification result of multiple indices and estimate which type of data is more valuable for the detection of OCD, we performed an automated classification with structural-MRI-derived GMV and WMV, along with DTI-derived FA and MD values in a large sample of OCD patients and matched HC. Ten brain regions that weighted the most in the identification of the four features were also calculated. To the best of our knowledge, this is the first study using SVM for classification of OCD patients based on whole-brain volumetry and tractography indices. We expected our study would be informative for the early detection of patients with OCD in future clinical applications.

## Materials and methods

### Subjects

This study was approved by the Ethics Review Board of Kunming Medical University. The written informed consent of each participant was obtained before the study.

In this study, 48 OCD patients were recruited from the psychiatry department of the First Affiliated Hospital of Kunming Medical University. One experienced clinical psychiatrist made the OCD diagnosis according to the Diagnostic and Statistical Manual of Mental Disorders-Fourth Edition (DSM-IV) criteria. Medication status of OCD sample were listed in the Supplementary Material (see, Table S1). Forty-five well-matched healthy control volunteers were recruited by advertisement. All the participants were right-handed Han Chinese individuals aged from 18 to 55 years. The exclusion criteria for participants involved in both groups were as below: (1) having a previous history of other psychiatric or neurological illness or serious physical disease, (2) present or previous history of substance abuse, (3) physical limitations to undergo an MRI scan, (4) pregnant women, (5) right handedness. Besides, all patients' obsessive-compulsive symptoms were not caused by another mental disorder or physical disease. Demographic data, including age, sex, duration of illness, and clinical symptom ratings were obtained. The Yale-Brown Obsessive-Compulsive Scale (Y-BOCS) was used to evaluate obsessive-compulsive symptoms. In consideration of the results might be influenced by comorbidity of depression and anxiety, the patients with elevated depression (The Hamilton Depression Rating Scale score, HDRS >17) or anxiety (Hamilton Anxiety Scale score, HAMA >14) symptoms were excluded.

Demographic and clinical characteristics differences between two groups were analyzed using IBM SPSS Statistics (version 21.0; IBM, Armonk, NY, USA). Age difference was assessed by the Independent samples *t*-test. Chi-Square test was performed to compare gender difference.

### Image acquisition

MRI scanning was carried out by a skilled radiological technician at a Philips Achieva 3.0T scanner (Philips Healthcare, Best, the Netherlands). First, acquisitions included a conventional normal T1- and T2-weighted sequences to rule out obvious structural abnormalities such as cerebrovascular diseases.

3D T1-weighted volumetric structural MRI scan sequence was acquired using a fast spoiled gradient recalled acquisition (FSPGR) with the following parameters: TR/TE = 7.38/3.4 ms, matrix size = 256 × 256, FOV = 250 × 250 mm, number of slices: 230, flip angle = 90°, scan time = 6 min 53 s.

DTI images were acquired using an echo-planar imaging (EPI) sequence in 50 axial planes and was collected along 33 independent orientations through the whole brain using the following parameters: TR/TE = 6,800 ms/80 ms, slice thickness = 3 mm, FOV = 230 mm2 × 230 mm2, matrix size = 116 × 112, voxel size = 1.98 mm × 2.05 mm × 3 mm, b value = 1000 s/mm2, flip angle = 90°.

### Image preprocessing

Structural MRI images were preprocessed using the VBM8 toolbox (http://dbm.neuro.uni-jena.de/vbm) with the Diffeomorphic Anatomical Registration using the Exponentiated Liealgebra (DARTEL) toolbox (37) implemented in the statistic parametric mapping software package (SPM8, http://www.fil.ion.ucl.ac.uk/spm) running on Matlab 2012a (MathWorks, Natick, MA, USA). This procedure comprises creating a study-specific template and segmenting each individual image using the template aiming to maximize accuracy and sensitivity (29). Then the GM, WM and cerebrospinal fluid (CSF) were automatically segmented. After Jacobian modulation, the GM images and WM images were smoothed with 8-mm full width at half maximum (FWHM) Gaussian kernel for further SVM analysis.

The diffusion MRI data were processed by the FMRIB Software Library (FSL, Version 5.0; available from http://fsl.fmrib.ox.ac.uk/fsl). Tract-based spatial statistics (TBSS) was used to perform voxel-wise processing of diffusivity measures. First, the skull was stripped using Brain Extraction Tool (BET) of FSL. Then the head motion and eddy current distortion correction were conducted using the *b* = 0 volume as a reference. FMRIB's Diffusion Toolbox (FDT) was used to fit the tensor model and to compute the FA and MD images using the standard FSL protocol similarly with the previous DTI studies (38–40). Then, the FA and MD of each subject were aligned to the FMRI58-FA template (http://fsl.fmrib.ox.ac.uk/fsl/fslwiki/FMRIB58_FA) by using the non-linear image registration tool (FNIRT). Subsequently, the mean FA and MD images in each WM tract were calculated for each subject. Then a mean FA skeleton was generated as the mean FA image was created by averaging all aligned FA maps, which represents the centers of all fiber tracts common to all subjects. A threshold of FA ≥0.2 was set to include the major white matter pathways. The MD maps were acquired using the same non-linear transformations as the FA maps.

### Support vector machine analysis

SVM was applied by using the Pattern Recognition for Neuroimaging Toolbox (PRoNTo) (41) (http://www.mlnl.cs.ucl.ac.uk/pronto) to estimate potential WM areas contribute most in calcifying OCD. Briefly, the main steps of the SVM method include: (i) feature extraction and feature selection, (ii) selecting discriminative regions, (iii) training the SVM classifier model using the training data, and (iv) evaluating the performance of the SVM model using the evaluation data (9, 38).

In neuroimaging studies, the number of features is much more than the number of subjects, which induces the “curse of dimensionality” in machine learning studies (42, 43). Feature extraction allows the original data to transform into analyzable input data of SVM. In the present study, each 3D image was transformed into a column vector of features and each value corresponded to a single corresponding voxel intensity. Thus, this feature vector encoded the pattern of GMV, WMV, FA, or MD values. By comparison, feature selection involves the selection of a subset of features which facilitates learning (15, 44). In this study, feature selection consisted of identifying brain regions that are expected to differ between groups (15, 44). Above procedures were automatically processed in PRoNTo's “Prepare feature set” programs.

A leave-one-out cross-validation method was carried out to perform SVM classifier validation, where the feature selection was performed each time on the training partition of the data to avoid circularity effects. In this study, it involved the exclusion of a single subject from each group and training the classifier using the remaining subjects. Then the excluded subject pair was used to test the ability of the classifier to classify new cases reliably. Above procedures were repeated for each subject pair so that it could obtain a relatively unbiased estimate of generalizability (44). Above procedures were automatically processed in PRoNTo's “Specify model” programs. And the whole process had been described in previous studies detailedly (24).

As for performance evaluation, once the SVM algorithm has been established, it is used to predict a new and previously unseen subject to and decide which group it belongs (44). A 1,000-times non-parametric permutation test (24, 28, 45, 46) was used to obtain a corrected *p-*value to determine the statistical significance of the accuracy, sensitivity and specificity. In detail, accuracy is the proportion of subjects correctly classified into the patient or control group. Sensitivity and specificity represent the proportion of subjects classified correctly. Besides, receiver operating characteristic (ROC) analysis and AUC (the area under the ROC curve) were used to evaluate the performance of the classifiers. AUC represents the classification power of a classifier, and a larger AUC indicates better classification ability (28, 47). This involves the repetitions of the classification procedure with training group labels by multiple random distribution of the computer in the aim of generating a null distribution of accuracies (44). We also performed a support vector regression of the magnitude of differences and illness duration.

For each model, the PRoNTo allows it possible to calculate images representing the weights per voxel and also images summarizing the weights per regions of interest as defined by an atlas (41). The region contributions can be ranked in descending order, yielding a sorted list of regions according to their contribution to the classification model. To investigate classification power of specific locations in the brain, we computed vector weights and listed 10 brain regions that have the highest value of the discrimination map of each feature. For the GMV feature, the most commonly used AAL atlas (48) was selected, which contains 116 cortical and subcortical anatomical structures. For the WMV and DTI feature sets, the weights were calculated based on the ICBM-DTI-81 white-matter atlas, containing 48 WM fiber tract labels (49).

## Result

### Demographic and clinical characteristics

The demographic and clinical characteristics of the subjects were presented in Table 1. No significant difference between groups was found in gender and age. The 48 OCD patients had an average duration of illness of 45.42 ± 41.02 months, the total Y-BOCS score was 25.50 ± 3.56, the Y-BOCS obsession score was 12.90 ± 2.40, the Y-BOCS compulsion score was 12.58 ± 3.07. The total HDRS and HAMA scores were 8.10 ± 3.71 and 9.29 ± 2.89, respectively (Table 1).

**Table 1:** Demographics and clinical characteristics of the sample.

	**OCD patients (*n* = 48)**	**HC (*n =* 45)**	***t/F/*χ2**	***p*-Value**
Age, years	32.29 ± 12.62	30.62 ± 9.02	4.733	0.464
Gender (male/female)	27/21	24/21	0.080	0.778
Duration (month)	45.42 ± 41.02	–	–	–
Y-BOCS total score	25.50 ± 3.56	–	–	–
Y-BOCS obsession score	12.90 ± 2.40	–	–	–
Y-BOCS compulsion score	12.58 ± 3.07	–	–	–
HDRS score	8.10 ± 3.71	–	–	–
HAMA score	9.29 ± 2.89	–	–	–

*OCD, obsessive-compulsive disorder; HC, Healthy Controls; Y-BOCS, Yale-Brown Obsessive-Compulsive Scale; HDRS, Hamilton Depression Rating Scale; HAMA, Hamilton Anxiety Scale*.

### Classifier evaluation

In the classification of the two groups, for the whole brain GMV, the accuracy was 72.04% (permutation *p* = 0.001) with a sensitivity of 70.83% and a specificity of 73.33%. For the whole brain WMV, the accuracy was 61.29% (permutation *p* = 0.040) with a sensitivity of 64.58% and a specificity of 57.78%. Results for the FA and MD values were more promising. A classification accuracy of 80.65 to 77.42% were achieved for the two feature sets of DTI (permutation *p* = 0.001), respectively. Sensitivity of the two parameters was 81.25 and 75%, respectively, and specificity of both FA and MD values were 80% (Table 2). No significant results were found between the four features and duration (see Table S3).

**Table 2:** SVM Classification performances of the four features.

	**Accuracy (%)**	**Sensitivity (%)**	**Specificity (%)**	**AUC**	**Permutation *P-*values**
GMV	72.04	70.83	73.33	0.71	0.001
WMV	61.29	64.58	57.78	0.61	0.040
FA	80.65	81.25	80.00	0.83	0.001
MD	77.42	75.00	80.00	0.84	0.001

*GMV, gray matter volume; WMV, white matter volume; FA, fractional anisotropy; MD, mean diffusivity; AUC, area under the ROC curve*.

**Figure 1:**
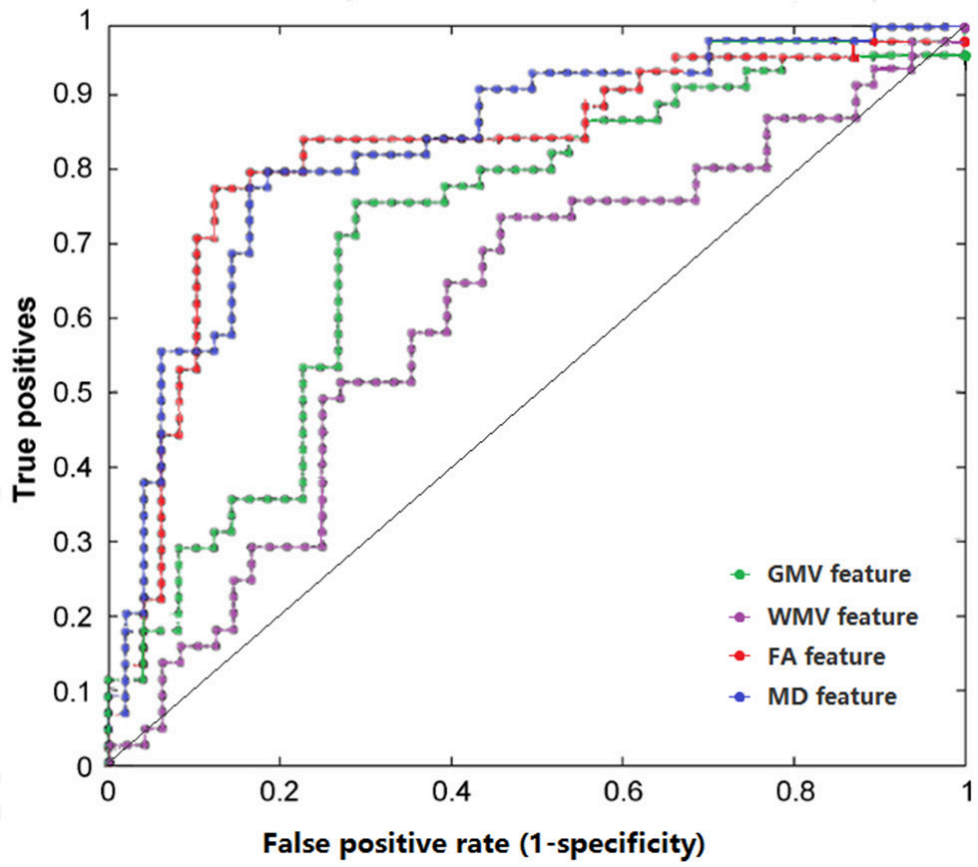
The ROC curves of classifier performance of distinct features. GMV, gray matter volume; WMV, white matter volume; FA, fractional anisotropy; MD, mean diffusivity.

Receiver Operating Characteristic (ROC) curve and area under the ROC (AUC) were also achieved (Figure 1, Table 2).

### Regions contributed most for classification

For GMV feature, the most informative regions for classification between OCD patients and HC included right anterior cingulate gyrus (ACG), right angular gyrus, right inferior parietal, bilateral paracentral lobule, left inferior frontal gyrus, and bilateral cerebelum regions. For the WMV feature and two feature sets of DTI, regions contributed the most to the discrimination were relatively consistent, mainly included the UNC, the cingulum in the hippocampus, corticospinal tract, as well as cerebellar peduncle. Additionally, right external capsule, left fornix and stria terminalis, left anterior corona radiate, bilateral cerebral peduncle, pontine crossing tract and bilateral cerebral peduncle are among the informative regions for classification (Table 3). (Detailed results could be obtained in Table S2).

**Table 3:** Ten brain regions contributed most for classification between OCD and control groups of the four features.

**Modality**	**Hemisphere L/R**	**Brain regions**	**ROI index**	**Discriminative weight (%)**	**Cluster Size**
**GMV**					
	L	Cerebelum 7b	101	1.677	1353
	L	Cerebelum 8	103	1.608	4504
	R	Cerebelum 7b	102	1.584	1233
	R	Angular gyrus	66	1.556	4097
	R	Cerebelum 8	104	1.442	5371
	R	Anterior cingulate gyrus	32	1.429	2996
	L	Paracentral lobule	69	1.424	3227
	R	Inferior parietal	62	1.392	3071
	L	Inferior frontal gyrus	11	1.340	2441
	R	Paracentral lobule	70	1.317	1944
**WMV**					
	R	Uncinate fasciculus	45	9.338	121
	R	Inferior cerebellar peduncle	11	5.660	291
	L	Inferior cerebellar peduncle	12	4.849	282
	R	Cingulum (hippocampus)	37	4.273	370
	L	Corticospinal tract	8	3.714	395
	L	Cingulum (hippocampus)	38	2.985	339
	R	External capsule	33	2.882	1609
	L	Anterior corona radiata	24	2.841	2035
	L	Uncinate fasciculus	46	2.595	111
	L	Fornix and stria terminalis	40	2.541	307
**FA**					
	L	Uncinate fasciculus	46	8.593	155
	R	Corticospinal tract	7	5.504	168
	R	Inferior cerebellar peduncle	11	4.861	134
	R	Cingulum (hippocampus)	37	4.355	172
	L	Corticospinal tract	8	4.254	164
		Pontine crossing tract	2	4.051	198
	L	Superior cerebellar peduncle	14	4.016	137
	L	Cerebral peduncle	16	3.643	312
	R	Cerebral peduncle	15	3.018	301
	L	Cingulum (hippocampus)	38	2.950	157
**MD**					
	L	Corticospinal tract	8	7.314	164
	R	Inferior cerebellar peduncle	11	6.507	134
	L	Inferior cerebellar peduncle	12	4.991	125
	R	Corticospinal tract	7	4.763	168
	R	Cingulum (hippocampus)	37	3.709	172
	R	Cerebral peduncle	15	3.459	301
		Pontine crossing tract	2	3.326	198
	L	Cerebral peduncle	16	3.276	312
	R	Superior cerebellar peduncle	13	3.268	128
	L	Cingulum (hippocampus)	38	3.182	157

*L, left; R, right. GMV feature was estimated using AAL atlas. WMV, FA, and MD features were estimated using ICBM-DTI-81 white-matter atlas*.

## Discussion

To the best of our knowledge, the present study is the first to simultaneously detect whole-brain volumetric and tractography abnormalities of OCD patients using SVM. Compared with the mass univariate analysis, SVM takes into account the inter-regional correlations, and provides numerical indicators for group membership without multiple comparison biases (44). We made this study aiming to develop an objective method facilitate the accuracy of the diagnosis and assist humans with clinical judgment. The primary finding of this preliminary study is that it is feasible to discriminate OCD patients from HC with high accuracies by using a neuroimaging-based computer-aided method.

According to the recent review of multivariate pattern analysis techniques on neuroimaging data of OCD (30), studies was able to classify OCD diagnosis with accuracies ranging from 66 up to 100%. Methodological limitations such as heterogeneity of sample characteristics, differences in data processing, acquisition or statistical analyses might induce these inconsistencies. In our study, the SVM analysis demonstrated better performance by using diffusion parameters than volumetric features for distinguishing OCD patients from HC. Among the four feature sets, FA came out to be the most significant one with a classification accuracy of 80.65%. This is close to the SVM classification on DTI-derived FA features performed by Li et al. (16). As FA and MD reflect white matter microstructure characteristics, our finding indicated that OCD patients might exhibited more detectable alterations in white matter integrity.

Our previous multimodal group-level study detected OCD patients exhibited reduced cortical thickness of the right posterior cingulate cortex and altered WM integrity in the genu and body of corpus callosum. Other multimodal imaging analyses reported OCD patients displayed alterations in brain structures and functions, involving complex brain networks (50–52). Multivariate methods provided a broader perspective to clarify the neuropathological mechanism of OCD. Nowadays SVM studies on OCD were mainly based on single modal. The current results showed that it is achievable to classify OCD patients and healthy people with multiple indices using an automatic machine learning way. The SVM algorithm calculates a region's discriminative power depends not only on between-group differences in its absolute values, but also on any between-group differences in its structural correlations with other regions. Therefore, this method is particularly suitable to explore mental disorders such as OCD in which abnormalities are distributed across the whole brain (16). These provided various perspectives for the investigation of pathological characteristics, as well as the detection of biomarkers for OCD diagnosis.

GM regions with high discriminative power are consistent with the previous structural MRI multivariate pattern analysis (31) and numerous VBM studies of OCD (1, 6), mainly distributed in the orbitofronto-striatal “affective” circuit, the DL prefronto-striatal “executive” circuit and the cerebellum (6). The cingulate cortex plays a key role in the “affective” circuit and ACG is considered to be involved in executive control, particularly of emotion-related processes (6, 53). Abnormal regional ACG volume has also been reported in previous VBM studies (54–57). The ACG volume deficit might mediate the cognitive symptoms (e.g., cognitive behavioral inflexibility) which were often observed in OCD patients (6). Previous functional MRI study in our research group also found that OCD patients exhibited decreased regional activity in the posterior cingulate cortex and increased activity in the ACG (58). Thus, characteristic morphometry changes of ACG might be a crucial biomarker of OCD. The right angular gyrus and right inferior parietal gyrus also showed a relatively high weight in the classification. The angular gyrus lies in the superior portion of the right lateral parietal cortex (59). Neuroimaging studies have reported abnormalities in the right parietal lobe, particularly in the angular gyrus (53, 60). Parietal lobe is important in a variety of executive tasks involving attention, spatial perception and working memory functions (53). Considering that impairment in some of these functions are consistently reported in OCD, such as attentional shifting, it is conceivable that parietal lobe dysfunction, particularly within the angular gyrus could contribute to the cognitive deficits evident in OCD (53, 61). In addition, the parietal lobe might interact with the frontal subcortical circuitry of OCD through direct anatomical connections between associative parietal areas and some of the key regions implicated in the disorder such as the lateral orbitofrontal cortex, the striatum and the mediodorsal thalamic nucleus (6). The paracentral lobule is divided into anterior and posterior to the central sulcus. The anterior portion of the paracentral lobule is part of the frontal lobe and is often referred to as the supplementary motor area; the posterior portion is considered part of the parietal lobe (62, 63). The function of parietal lobe has been proved to be primarily associated with skill learning, attention, and working memory (63). Therefore, in addition to the ACG, the frontal lobe and parietal lobe, paracentral lobule may also play key roles in the pathology of OCD and can provide much reference for classification of the disease.

Myelin is closely related with the complex connectivity of the human brain. Notably, in this study, three distinct WM classification models (WMV, FA, and MD) revealed a coherent set of discriminative features, which were primarily associated with processes of decision making or behavior control. UNC and cingulum are within the association fiber tracts (49). The UNC connects the frontal lobe (orbital cortex) and the anterior temporal lobe, while cingulum especially the hippocampal regions carries information from the cingulate gyrus to the hippocampus (49). The corticospinal tract is an important part of the motor system, and its integrity change has been widely detected in various studies (4). Disrupted white matter connectivity in these regions might underlie the neural mechanisms of decline in memory and behavior control in OCD patients. Since both the WM volumetric feature and the tractography indices indicated high discriminative power in the UNC, the cingulum in the hippocampus and corticospinal tract, we hypothesized that the above three regions (the UNC, the cingulum in the hippocampus and corticospinal tract) hold the most discriminative WM connections of OCD and are likely to be the specific biological markers of OCD. Additionally, other discovered informative white matter regions of the right external capsule, left fornix, and stria terminalis are parts of the association fibers (cortex-cortex connections), and the left anterior corona radiate and bilateral cerebral peduncle are defined as projection fibers (cortex–spinal cord, and cortex-thalamus connections) (49, 64). Damages of these WM tracts might interfere with the connectivity between brain regions and disrupt the brain networks involved in mood and cognition. These regions might provide informative value for detection of OCD as well.

It is particularly noteworthy that cerebellar regions showed a fairly high discriminative weight in all four features. This finding is in line with a previous structural study using the multivariate pattern analysis in OCD (31). Existing structural and functional neuroimaging studies have observed abnormal structures and functions in the cerebellum of OCD patients (65) and enriched our understanding of the great role of cerebellum in OCD illness models. Increasing evidence has demonstrated that in addition to its well-known role in motor control, the cerebellum also plays roles in cognitive and emotional regulatory processes (31, 66). The cerebellum is structurally and functionally connected to the parallel cortico-striato-thalamo-cortical circuits, as well as the limbic-cortical network (31, 67), which forms a feedback information flow that allows the cerebellum to involve in advanced neural activities. Moreover, abnormal cerebellar functions are suggested to be related to inflexible movements and repetitive behaviors in OCD (68). Hence, the cerebellum deserves more attention in future pathological model of OCD, and our results provide further evidence for neural implications of this region in OCD.

There are some limitations in this study to be addressed. First, this study aims not only to use multi-modal features for OCD classification, but also estimates which type of data is more valuable for the detection of OCD. So, this study did not investigate combined imaging modalities for classification. Multi-kernel learning based on multi-modal features with large sample might provide a better perspective to classify OCD from healthy controls in future studies. Second, our patients group consisted of both medicated and drug-naïve subjects. Medication status of the OCD group were various and complicated. Correlation between medication status and brain images was not analyzed separately. Therefore, we did not clarify how medication status would affect the brain. Last but not least, we innovatively estimate the 10 most discriminative brain regions to exhibit a preliminary result of the multiple feature sets. Some unlisted regions may also contain valuable discriminative information and these regions should receive full considerations in future research.

## Conclusion

Based on whole-brain volumetry and DTI images, SVM algorithm revealed high accuracies for distinguishing OCD patients from healthy controls at the level of the individual. Feature sets of DTI seem to offer better predictive value than volumetric features. Though machine learning method sole is not enough to diagnose the OCD patient than the clinical symptom, we believe neuroimaging-based machine learning techniques may suggest neurobiological markers for automatic detection of OCD patients and develop an objective method to facilitate the accuracy of the diagnosis. We hope computer-aided method based on imaging biomarker could assist clinicians with the diagnosis of mental disorders in the future.

## Ethics statement

This study was approved by the Ethics Review Board of Kunming Medical University (ClinicalTrials.-gov: NCT01298622). All of the subjects involved in the study gave written informed consent in accordance with the Declaration of Helsinki.

## Author contributions

XX designed the study and revised the manuscript. CZ, YC, LP, JX, ZS, LS, and SY carried out experiments and undertook the statistical analysis. LJ managed the diagnosis and clinical measurement. CZ wrote the first draft of manuscript. YL were responsible for acquiring MRI image data and assisted with the analysis. YC and LP contributed to the final manuscript.

### Conflict of interest statement

The authors declare that the research was conducted in the absence of any commercial or financial relationships that could be construed as a potential conflict of interest.
