# Supplementary material for: Support Vector Machine Classification of Obsessive-Compulsive Disorder Based on Whole-Brain Volumetry and Diffusion Tensor Imaging
Source: Front Psychiatry. 2018 Oct 23;9:524. doi: 10.3389/fpsyt.2018.00524 (PMC6206075; doi:10.3389/fpsyt.2018.00524)
Supplement: Supplementary file 1 [file Table_1.DOC]

**Supplementary Material**

**Table S1** Medication status of OCD sample (n=48)

| Medication Status | *N* | Dosage (/day) |
| --- | --- | --- |
| No medication | 25 (52.08) | - |
| Antidepressant | 22 (45.83) | - |
| Clomipramine | 3 | 50-200 mg |
| Fluxomanine | 3 | 100-200 mg |
| Fluoxetine | 5 | 40-80 mg |
| Sertraline | 6 | 50-100 mg |
| Paroxetine | 5 | 40-60 mg |
| Benzodiazepine | 0 | - |
| Antidepressant + Benzodiazepine | 1 (2.08) | Paroxetine 40 mg + Estazolam 1 mg |

**Table S2** Brain regions contributing for classification between OCD and control groups

| Modality | Brain regions | ROI index | Discriminative weight (%) | Cluster Size |
| --- | --- | --- | --- | --- |
| GMV |  |  |  |  |
|  | Cerebelum_7b_L | 101 | 1.6771 | 1353 |
|  | Cerebelum_8_L | 103 | 1.6077 | 4504 |
|  | Cerebelum_7b_R | 102 | 1.584 | 1233 |
|  | Angular_R | 66 | 1.5558 | 4097 |
|  | Cerebelum_8_R | 104 | 1.4416 | 5371 |
|  | Cingulum_Ant_R | 32 | 1.429 | 2996 |
|  | Paracentral_Lobule_L | 69 | 1.4242 | 3227 |
|  | Parietal_Inf_R | 62 | 1.3919 | 3071 |
|  | Frontal_Inf_Oper_L | 11 | 1.3396 | 2441 |
|  | Paracentral_Lobule_R | 70 | 1.3166 | 1944 |
|  | Cingulum_Post_L | 35 | 1.3087 | 1132 |
|  | Cerebelum_3_L | 95 | 1.2999 | 320 |
|  | Cingulum_Post_R | 36 | 1.2856 | 745 |
|  | Angular_L | 65 | 1.2736 | 2763 |
|  | Cuneus_L | 45 | 1.2655 | 3611 |
|  | Postcentral_R | 58 | 1.2254 | 9077 |
|  | Cerebelum_9_R | 106 | 1.2066 | 1900 |
|  | Fusiform_R | 56 | 1.2059 | 6012 |
|  | Parietal_Sup_L | 59 | 1.1439 | 4940 |
|  | Precuneus_R | 68 | 1.129 | 7521 |
|  | Cingulum_Mid_R | 34 | 1.129 | 5117 |
|  | Heschl_R | 80 | 1.1172 | 580 |
|  | Temporal_Inf_R | 90 | 1.1052 | 8573 |
|  | Cingulum_Ant_L | 31 | 1.0934 | 3384 |
|  | Precuneus_L | 67 | 1.0813 | 8481 |
|  | Cerebelum_Crus2_L | 93 | 1.0774 | 4589 |
|  | Parietal_Sup_R | 60 | 1.0463 | 5104 |
|  | Vermis_3 | 110 | 1.0382 | 570 |
|  | Parietal_Inf_L | 61 | 1.0233 | 5765 |
|  | Precentral_R | 2 | 1.0196 | 8098 |
|  | Occipital_Mid_L | 51 | 1.0164 | 7646 |
|  | Postcentral_L | 57 | 1.0089 | 9252 |
|  | Vermis_1_2 | 109 | 0.9949 | 118 |
|  | Cerebelum_3_R | 96 | 0.9792 | 461 |
|  | Supp_Motor_Area_R | 20 | 0.9669 | 5401 |
|  | Cerebelum_9_L | 105 | 0.9603 | 2044 |
|  | Occipital_Sup_R | 50 | 0.9559 | 3233 |
|  | Frontal_Sup_R    Frontal_Sup_R | 4 | 0.9531 | 9341 |
|  | Occipital_Inf_L | 53 | 0.9501 | 2233 |
|  | Temporal_Sup_L | 81 | 0.9384 | 5304 |
|  | Vermis_4_5 | 111 | 0.9367 | 1633 |
|  | Temporal_Sup_R | 82 | 0.9358 | 7440 |
|  | SupraMarginal_R | 64 | 0.9354 | 4687 |
|  | Occipital_Sup_L | 49 | 0.9329 | 3177 |
|  | Occipital_Mid_R | 52 | 0.9304 | 4897 |
|  | Temporal_Inf_L | 89 | 0.9251 | 7613 |
|  | Cerebelum_Crus2_R | 94 | 0.9135 | 5116 |
|  | Cingulum_Mid_L | 33 | 0.9118 | 4749 |
|  | Hippocampus_R | 38 | 0.9067 | 2223 |
|  | Supp_Motor_Area_L | 19 | 0.8995 | 5205 |
|  | Cuneus_R | 46 | 0.891 | 3512 |
|  | Frontal_Inf_Oper_R | 12 | 0.8815 | 3333 |
|  | Temporal_Mid_L | 85 | 0.8801 | 11680 |
|  | Lingual_R | 48 | 0.8724 | 5501 |
|  | Calcarine_L | 43 | 0.8589 | 5220 |
|  | Insula_R | 30 | 0.8542 | 4169 |
|  | Precentral_L | 1 | 0.8537 | 8387 |
|  | Fusiform_L | 55 | 0.8495 | 5471 |
|  | Temporal_Pole_Mid_L | 87 | 0.8382 | 1764 |
|  | Temporal_Mid_R | 86 | 0.8256 | 10501 |
|  | Vermis_9 | 115 | 0.8206 | 404 |
|  | Cerebelum_4_5_R | 98 | 0.8082 | 1958 |
|  | Putamen_L | 73 | 0.8052 | 2357 |
|  | Frontal_Inf_Tri_R | 14 | 0.7966 | 4945 |
|  | Frontal_Mid_R | 8 | 0.7952 | 11916 |
|  | Cerebelum_4_5_L | 97 | 0.7942 | 2633 |
|  | Frontal_Inf_Orb_R | 16 | 0.7693 | 4085 |
|  | Vermis_8 | 114 | 0.7655 | 566 |
|  | Frontal_Sup_L | 3 | 0.7603 | 8607 |
|  | Calcarine_R | 44 | 0.7368 | 4291 |
|  | Cerebelum_10_R | 108 | 0.7338 | 364 |
|  | Cerebelum_Crus1_L | 91 | 0.7292 | 6113 |
|  | Frontal_Mid_L | 7 | 0.7227 | 11528 |
|  | Olfactory_R | 22 | 0.7224 | 651 |
|  | Frontal_Mid_Orb_L | 9 | 0.708 | 2134 |
|  | Temporal_Pole_Mid_R | 88 | 0.7079 | 2808 |
|  | Vermis_7 | 113 | 0.7038 | 463 |
|  | Cerebelum_Crus1_R | 92 | 0.693 | 6191 |
|  | Putamen_R | 74 | 0.6857 | 2529 |
|  | Rolandic_Oper_L | 17 | 0.68 | 2379 |
|  | SupraMarginal_L | 63 | 0.6781 | 2909 |
|  | Cerebelum_6_L | 99 | 0.6755 | 4034 |
|  | Frontal_Mid_Orb_L | 25 | 0.6749 | 1676 |
|  | Frontal_Inf_Orb_L | 15 | 0.6674 | 4140 |
|  | Rolandic_Oper_R | 18 | 0.6606 | 3143 |
|  | Insula_L | 29 | 0.6604 | 4400 |
|  | Frontal_Sup_Orb_R | 6 | 0.6601 | 2312 |
|  | Lingual_L | 47 | 0.6503 | 5168 |
|  | ParaHippocampal_R | 40 | 0.6468 | 2673 |
|  | Occipital_Inf_R | 54 | 0.6454 | 2385 |
|  | Amygdala_L | 41 | 0.639 | 522 |
|  | Rectus_R | 28 | 0.6321 | 1741 |
|  | Frontal_Mid_Orb_R | 26 | 0.612 | 1976 |
|  | Hippocampus_L | 37 | 0.611 | 2199 |
|  | Frontal_Sup_Medial_L | 23 | 0.5901 | 7016 |
|  | Cerebelum_6_R | 100 | 0.5854 | 4229 |
|  | Frontal_Inf_Tri_L | 13 | 0.5786 | 5863 |
|  | Vermis_6 | 112 | 0.5607 | 901 |
|  | ParaHippocampal_L | 39 | 0.5589 | 2336 |
|  | Temporal_Pole_Sup_L | 83 | 0.5438 | 3053 |
|  | Frontal_Sup_Orb_L | 5 | 0.5226 | 2163 |
|  | Frontal_Sup_Medial_R | 24 | 0.5217 | 5117 |
|  | Pallidum_L | 75 | 0.4941 | 692 |
|  | Thalamus_R | 78 | 0.4873 | 2409 |
|  | Heschl_L | 79 | 0.4823 | 535 |
|  | Frontal_Mid_Orb_R | 10 | 0.4816 | 2380 |
|  | Thalamus_L | 77 | 0.4726 | 2479 |
|  | Caudate_R | 72 | 0.4677 | 2314 |
|  | Olfactory_L | 21 | 0.4608 | 666 |
|  | Amygdala_R | 42 | 0.4542 | 577 |
|  | Temporal_Pole_Sup_R | 84 | 0.439 | 3133 |
|  | Rectus_L | 27 | 0.4375 | 2127 |
|  | Caudate_L | 71 | 0.391 | 2295 |
|  | Pallidum_R | 76 | 0.3656 | 645 |
|  | Vermis_10 | 116 | 0.3557 | 265 |
|  |  | 107 | 0.2976 | 340 |
| WMV |  |  |  |  |
|  | Uncinate fasciculus R | 45 | 9.3318 | 121 |
|  | Inferior cerebellar peduncle R | 11 | 5.6601 | 291 |
|  | Inferior cerebellar peduncle L | 12 | 4.849 | 282 |
|  | Cingulum (hippocampus) R | 37 | 4.2725 | 370 |
|  | Corticospinal tract L | 8 | 3.714 | 395 |
|  | Cingulum (hippocampus) L | 38 | 2.9846 | 339 |
|  | External capsule R | 33 | 2.8824 | 1609 |
|  | Anterior corona radiata L | 24 | 2.8411 | 2035 |
|  | Uncinate fasciculus L | 46 | 2.5947 | 111 |
|  | Fornix (cres) / Stria terminalis L | 40 | 2.5406 | 307 |
|  | Sagittal stratum R | 31 | 2.4911 | 666 |
|  | Superior cerebellar peduncle R | 13 | 2.4183 | 279 |
|  | Superior cerebellar peduncle L | 14 | 2.0721 | 280 |
|  | Corticospinal tract R | 7 | 2.0212 | 403 |
|  | Fornix (cres) / Stria terminalis R | 39 | 2.0097 | 333 |
|  | Middle cerebellar peduncle | 1 | 1.9932 | 4575 |
|  | Pontine crossing tract (a part of MCP) | 2 | 1.9911 | 421 |
|  | Cerebral peduncle L | 16 | 1.9727 | 653 |
|  | Cingulum (cingulate gyrus) L | 36 | 1.9699 | 858 |
|  | Cerebral peduncle R | 15 | 1.9278 | 659 |
|  | Superior corona radiata L | 26 | 1.8839 | 2184 |
|  | Posterior thalamic radiation L | 30 | 1.8279 | 1236 |
|  | Medial lemniscus L | 10 | 1.7227 | 204 |
|  | Cingulum (cingulate gyrus) R | 35 | 1.7209 | 704 |
|  | Superior longitudinal fasciculus L | 42 | 1.6495 | 1933 |
|  | Tapetum R | 47 | 1.6444 | 179 |
|  | Superior longitudinal fasciculus R | 41 | 1.6137 | 1927 |
|  | Sagittal stratum L | 32 | 1.5747 | 638 |
|  | Superior corona radiata R | 25 | 1.5594 | 2135 |
|  | Posterior corona radiata R | 27 | 1.5289 | 1093 |
|  | External capsule L | 34 | 1.5209 | 1617 |
|  | Splenium of corpus callosum | 5 | 1.464 | 3736 |
|  | Posterior thalamic radiation R | 29 | 1.4372 | 1226 |
|  | Anterior corona radiata R | 23 | 1.3497 | 2035 |
|  | Posterior corona radiata L | 28 | 1.3071 | 1084 |
|  | Posterior limb of internal capsule L | 20 | 1.2588 | 1157 |
|  | Genu of corpus callosum | 3 | 1.1895 | 2668 |
|  | Anterior limb of internal capsule L | 18 | 1.1854 | 895 |
|  | Medial lemniscus R | 9 | 1.1463 | 197 |
|  | Fornix (column and body of fornix) | 6 | 1.1229 | 181 |
|  | Anterior limb of internal capsule R | 17 | 1.1226 | 957 |
|  | Retrolenticular part of internal capsule R | 21 | 1.1122 | 752 |
|  | Body of corpus callosum | 4 | 1.1084 | 4051 |
|  | Retrolenticular part of internal capsule L | 22 | 1.0254 | 732 |
|  | Posterior limb of internal capsule R | 19 | 0.953 | 1154 |
|  | Tapetum L | 48 | 0.8675 | 169 |
|  | Superior fronto-occipital fasciculus L | 44 | 0.79 | 122 |
|  | Superior fronto-occipital fasciculus R | 43 | 0.7751 | 119 |
| FA |  |  |  |  |
|  | Uncinate fasciculus L | 46 | 8.5932 | 155 |
|  | Corticospinal tract R | 7 | 5.5035 | 168 |
|  | Inferior cerebellar peduncle R | 11 | 4.8609 | 134 |
|  | Cingulum (hippocampus) R | 37 | 4.3547 | 172 |
|  | Corticospinal tract L | 8 | 4.2542 | 164 |
|  | Pontine crossing tract (a part of MCP) | 2 | 4.0514 | 198 |
|  | Superior cerebellar peduncle L | 14 | 4.0155 | 137 |
|  | Cerebral peduncle L | 16 | 3.6425 | 312 |
|  | Cerebral peduncle R | 15 | 3.0177 | 301 |
|  | Cingulum (hippocampus) L | 38 | 2.9497 | 157 |
|  | Middle cerebellar peduncle | 1 | 2.8707 | 1997 |
|  | Medial lemniscus R | 9 | 2.7506 | 92 |
|  | Inferior cerebellar peduncle L | 12 | 2.5834 | 125 |
|  | Uncinate fasciculus R | 45 | 2.4565 | 61 |
|  | Superior cerebellar peduncle R | 13 | 2.1632 | 128 |
|  | Medial lemniscus L | 10 | 1.9481 | 89 |
|  | Cingulum (cingulate gyrus) R | 35 | 1.882 | 302 |
|  | Tapetum R | 47 | 1.814 | 71 |
|  | Fornix (cres) / Stria terminalis R | 39 | 1.8137 | 146 |
|  | Cingulum (cingulate gyrus) L | 36 | 1.8005 | 374 |
|  | Sagittal stratum L | 32 | 1.7854 | 271 |
|  | External capsule L | 34 | 1.7397 | 746 |
|  | Posterior limb of internal capsule R | 19 | 1.698 | 512 |
|  | Fornix (cres) / Stria terminalis L | 40 | 1.6804 | 142 |
|  | Posterior thalamic radiation R | 29 | 1.4707 | 548 |
|  | Sagittal stratum R | 31 | 1.4159 | 266 |
|  | Posterior thalamic radiation L | 30 | 1.3956 | 562 |
|  | External capsule R | 33 | 1.3759 | 747 |
|  | Retrolenticular part of internal capsule R | 21 | 1.3045 | 359 |
|  | Splenium of corpus callosum | 5 | 1.3021 | 1642 |
|  | Anterior corona radiata L | 24 | 1.2862 | 905 |
|  | Anterior corona radiata R | 23 | 1.2326 | 905 |
|  | Superior corona radiata R | 25 | 1.2218 | 947 |
|  | Posterior limb of internal capsule L | 20 | 1.1917 | 503 |
|  | Body of corpus callosum | 4 | 1.151 | 1773 |
|  | Retrolenticular part of internal capsule L | 22 | 1.1436 | 333 |
|  | Posterior corona radiata L | 28 | 1.1335 | 469 |
|  | Genu of corpus callosum | 3 | 1.1327 | 1156 |
|  | Anterior limb of internal capsule L | 18 | 1.0973 | 411 |
|  | Superior longitudinal fasciculus R | 41 | 1.0829 | 849 |
|  | Posterior corona radiata R | 27 | 1.0406 | 492 |
|  | Superior corona radiata L | 26 | 0.9529 | 939 |
|  | Superior longitudinal fasciculus L | 42 | 0.9237 | 854 |
|  | Anterior limb of internal capsule R | 17 | 0.7669 | 418 |
|  | Fornix (column and body of fornix) | 6 | 0.7462 | 71 |
|  | Tapetum L | 48 | 0.7401 | 84 |
|  | Superior fronto-occipital fasciculus R | 43 | 0.6622 | 53 |
|  | Superior fronto-occipital fasciculus L | 44 | NA | 0 |
| MD |  |  |  |  |
|  | Corticospinal tract L | 8 | 7.3142 | 164 |
|  | Inferior cerebellar peduncle R | 11 | 6.507 | 134 |
|  | Inferior cerebellar peduncle L | 12 | 4.991 | 125 |
|  | Corticospinal tract R | 7 | 4.7634 | 168 |
|  | Cingulum (hippocampus) R | 37 | 3.7088 | 172 |
|  | Cerebral peduncle R | 15 | 3.4592 | 301 |
|  | Pontine crossing tract (a part of MCP) | 2 | 3.3263 | 198 |
|  | Cerebral peduncle L | 16 | 3.2763 | 312 |
|  | Superior cerebellar peduncle R | 13 | 3.2675 | 128 |
|  | Cingulum (hippocampus) L | 38 | 3.1818 | 157 |
|  | Medial lemniscus L | 10 | 2.6843 | 89 |
|  | Fornix (cres) / Stria terminalis L | 40 | 2.6376 | 142 |
|  | Retrolenticular part of internal capsule R | 21 | 2.597 | 359 |
|  | Uncinate fasciculus L | 46 | 2.2522 | 64 |
|  | Cingulum (cingulate gyrus) L | 36 | 2.0703 | 374 |
|  | Superior cerebellar peduncle L | 14 | 2.0006 | 137 |
|  | Uncinate fasciculus R | 45 | 1.9749 | 61 |
|  | Fornix (column and body of fornix) | 6 | 1.9715 | 71 |
|  | Fornix (cres) / Stria terminalis R | 39 | 1.955 | 146 |
|  | Medial lemniscus R | 9 | 1.9278 | 92 |
|  | Posterior limb of internal capsule R | 19 | 1.8929 | 512 |
|  | Genu of corpus callosum | 3 | 1.8191 | 1156 |
|  | Anterior limb of internal capsule R | 17 | 1.7968 | 418 |
|  | Body of corpus callosum | 4 | 1.7497 | 1773 |
|  | Cingulum (cingulate gyrus) R | 35 | 1.7021 | 302 |
|  | Middle cerebellar peduncle | 1 | 1.6872 | 1997 |
|  | Retrolenticular part of internal capsule L | 22 | 1.6132 | 333 |
|  | Anterior corona radiata L | 24 | 1.572 | 905 |
|  | Splenium of corpus callosum | 5 | 1.5547 | 1642 |
|  | Superior corona radiata R | 25 | 1.5168 | 947 |
|  | Sagittal stratum L | 32 | 1.4245 | 271 |
|  | External capsule R | 33 | 1.3946 | 747 |
|  | Anterior corona radiata R | 23 | 1.3329 | 905 |
|  | Sagittal stratum R | 31 | 1.3313 | 266 |
|  | Tapetum R | 47 | 1.3259 | 71 |
|  | External capsule L | 34 | 1.2887 | 746 |
|  | Posterior limb of internal capsule L | 20 | 1.1413 | 503 |
|  | Posterior corona radiata R | 27 | 1.0261 | 492 |
|  | Posterior thalamic radiation R | 29 | 0.9943 | 548 |
|  | Anterior limb of internal capsule L | 18 | 0.9428 | 411 |
|  | Tapetum L | 48 | 0.8046 | 84 |
|  | Superior fronto-occipital fasciculus R | 43 | 0.7756 | 53 |
|  | Superior longitudinal fasciculus L | 42 | 0.7215 | 854 |
|  | Superior corona radiata L | 26 | 0.6689 | 939 |
|  | Posterior thalamic radiation L | 30 | 0.6603 | 562 |
|  | Superior longitudinal fasciculus R | 41 | 0.6317 | 849 |
|  | Posterior corona radiata L | 28 | 0.516 | 469 |
|  | Superior fronto-occipital fasciculus L | 44 | 0.2477 | 48 |

L, left; R, right.

GMV feature was estimated using AAL atlas. WMV, FA and MD features were estimated using ICBM-DTI-81 white-matter atlas.

**Table S3** SVM regression performances of the magnitude of differences and illness duration.

| Features | *r* values | permutation *P* values |
| --- | --- | --- |
| GMV | -0.21 | 0.781 |
| WMV | -0.08 | 0.558 |
| FA | -0.01 | 0.399 |
| MD | 0.03 | 0.254 |

GMV, gray matter volume. WMV, white matter volume; FA, fractional anisotropy; MD, mean diffusivity.
